# Efficacy of Recombinant Human Bone Morphogenetic Protein-2 in Alveolar Cleft Treatment for Children: Systematic Review and Meta-Analysis

**DOI:** 10.3390/life15020185

**Published:** 2025-01-26

**Authors:** Ebtihal Ali A. Alawami, Fouad Alomari, Sarah A. Aloqaybi, Qusay Aloweiny, Lina Khalid Alswayed, Narjes W. Alshafai, Rawan Alhelal, Moayad M. Alfuraydi, Abdulaziz Fahad Samandar, Renad Abdulaziz Saleh Alsaeed, Danah Aldulaijan

**Affiliations:** 1 College of Dentistry, Vision Colleges, Riyadh 13226, Saudi Arabia; 2 Maxillofacial Surgeon, Department of Maxillofacial Surgery and Diagnostic Sciences, King Faisal Medical City, Abha 62586, Saudi Arabia; 3 College of Medicine, Bisha University, Bisha 67614, Saudi Arabia; sab825510@gmail.com; 4 College of Medicine, King Saud Bin Abdulaziz University for Health and Sciences, Jeddah 22384, Saudi Arabia; qusaya107@gmail.com; 5 College of Medicine, King Saud Bin Abdulaziz University for Health and Sciences, Riyadh 11481, Saudi Arabia; linakalswayed@gmail.com (L.K.A.); alhelalrawan@outlook.com (R.A.); 6 College of Dentistry, Imam Abdulrahman Bin Faisal University, Dammam 31441, Saudi Arabia; narjeswalshafai@gmail.com; 7 College of Dentistry, University of Hail, Hail 55475, Saudi Arabia; alferendo51@gmail.com; 8 College of Medicine, Umm-Al-Qura University, Makkah 21955, Saudi Arabia; ezoofahad@gmail.com; 9 College of Dentistry, Princess Nourah Bint Abdulrahman University, Riyadh 11671, Saudi Arabia; renadalsaeed9@hotmail.com; 10 College of Medicine, Imam Abdulrahman Bin Faisal University, Dammam 31441, Saudi Arabia; 2210001024@iau.edu.sa

**Keywords:** cleft lip/palate, iliac bone graft, rhBMP-2, bone morphogenic protein, absorbable collagen sponge, GRADE certainty of evidence

## Abstract

Background: Alveolar bone reconstruction with recombinant protein has several advantages, including less surgical timing, and reduced infection. This systematic review aims to assess the efficacy of recombinant human bone morphogenetic protein-2 (rhBMP-2) as a treatment modality for children with cleft lip and palate compared to the conventional iliac crest bone grafting approach. Methods: For current systematic review and meta-analysis, five electronic databases, namely, MEDLINE/PubMed, the Cochrane Central Register of Controlled Trials (CENTRAL), ClinicalTrials.gov, Web of Science, and ScienceDirect, were searched. The primary outcome measured in this review was bone volume and height after alveolar bone reconstruction surgery. The Risk of Bias Tool 2 assessed the risk of bias for randomized control trials and the Risk of Bias tool for non-randomized trials of interventions for non-randomized studies. By evaluating pooled meta-analysis, the mean difference was calculated. GRADE uncertainty of evidence was performed to assess the certainty of the results. Results: Of 230 identified studies, 6 randomized and 2 non-randomized studies were included in the current review. The average bone volume was higher among the rhBMP-2 group at 61.11% ± 24.6% than the iliac crest group at 59.12% ± 18.59%. The calculated mean bone height was higher in the iliac crest group at 78.65% ± 14.38% than in the rhBMP-2 group at 67.5% ± 5.45%. The risk of bias reported in the studies was low to moderate. The result of the meta-analysis supported using rhBMP-2 in alveolar bone reconstruction; however, no significant association was found (mean difference: −1.24; confidence interval: −4.14 to 1.67). Conclusions: The calculated meta-analysis reported no significant difference, and the quality of evidence measured was also moderate. Hence, more clinical trials are required to support using rhBMP-2 as an alternative to traditional techniques for treating cleft lip and palate.

## 1. Introduction

Cleft lip and palate are among the most common craniofacial disorders, affecting approximately 25% of children globally, with around 75% experiencing unilateral alveolar clefts [1]. An alveolar cleft refers to the gap in the maxillary anterior region extending to the incisive foramen, which disrupts the normal eruption of maxillary anterior teeth [2]. Surgical interventions aim to correct this condition and support orthodontic tooth movements, facilitate maxillary tooth eruption, improve speech, and provide structural support to the base of the nose [2]. Historically, iliac crest bone grafting has been considered the gold standard for alveolar reconstruction surgery [2]. The success rate of this procedure is well-documented, ranging from 50% to 80%, depending on donor site morbidity [3]. However, side effects such as pain at the donor site, compromised ambulation, and infection highlight the need for alternative approaches [4]. Moreover, to improve surgical outcomes and reduce morbidity, oral surgeons introduced bioactive materials that can improve the osteogenesis rate of the alveolar graft.

Studies have shown that recombinant human bone morphogenetic protein-2 (rhBMP-2) exhibits chemoattraction towards osteoprogenitor and stem cells, which serve as a bone-forming agent [5,6]. Platelet-rich plasma (PRP) is recognized for its abundance of growth factors and cell adhesion molecules, including fibrin, fibronectin, and vitronectin [6,7]. The interaction of these components exemplifies the tissue engineering triangle, highlighting the constructive collaboration between biological cues, scaffolds, and cellular elements in regenerative processes.

Clinical and preclinical studies demonstrated the potential of recombinant human bone morphogenetic protein-2 (rhBMP-2) as an alternative to iliac bone grafts [2,4,5]. Various advantages of this technique have been reported in the literature, including the absence of donor site complications, reduced operating time, and shorter hospitalization periods [2,4,5]. The first study on alveolar bone reconstruction using rhBMP-2 was conducted by Chin et al., who reported successful osseous union in 49 maxillary sites, supporting the use of rhBMP-2 for alveolar bone reconstruction [6]. Since then, several studies explored using absorbable collagen sponges (ACS), hyaluronan-based hydrogel, or demineralized bone matrix (DBM) scaffolds for rhBMP-2.

Dickinson et al. found that rhBMP-2 and resorbable collagen matrix improved bone growth in skeletally mature patients for alveolar cleft reconstruction. In the BMP-2 group, alveolar cleft filling was 95%, compared to 63% in the control group obtaining a standard iliac bone graft [7]. Recent investigations found no significant difference in alveolar cleft filling between ICBG and rhBMP-2/carrier groups [8,9,10]. In 2019, Jahanbin et al. conducted a study on alveolar cleft reconstruction utilizing autologous bone grafts with a low dose of rhBMP-2 (0.25 mg) [9]. Improved bone graft density and height were found three months following surgery in the rhBMP-2 group compared to the control group. Therefore, combining rhBMP-2 with autologous bone grafting presents a promising approach for achieving successful alveolar reconstruction in patients with cleft lip and palate.

Controversies persist regarding the ideal material for alveolar reconstruction surgeries. While various studies support using rhBMP-2 with scaffold materials, the iliac crest bone graft has historically been considered the gold standard for these procedures. A systematic review of the efficacy of rhBMP-2 highlighted a lack of sufficient evidence to fully support its use as a bioactive material [11]. This review focused exclusively on randomized controlled trials comparing rhBMP-2 with iliac bone grafts in alveolar reconstruction [11].

The current systematic review aims to evaluate the efficacy of rhBMP-2 in alveolar reconstruction by incorporating evidence from randomized controlled trials and observational studies. Specifically, the objective is to assess the effectiveness of rhBMP-2 as an alternative to iliac crest grafts in terms of bone filling and height. The hypothesis drawn for this systematic review was that there is no difference in the efficacy of the rhBMP-2 when compared to iliac bone graft in alveolar reconstruction.

## 2. Materials and Methods

### 2.1. Research Question and Protocol

Before the commencement of this review, the protocol was developed and registered on PROSPERO (CRD42023454690). The current systematic review was reported according to the guidelines developed by Preferred Reporting Items for Systematic Review and Meta-Analysis (PRISMA) [12]. The research question developed for this review was “In children with alveolar cleft defects, how does the use of Bone Morphogenetic Protein-2 (BMP-2) compared to Iliac crest bone grafting techniques help in achieving successful bone filling, volume, and bone height?” Where population: children with the alveolar cleft defect; intervention: bone morphogenetic protein-2 (BMP-2); comparison: iliac crest bone grafting; and outcome: bone filling, volume, and height.

### 2.2. Inclusion and Exclusion Criteria

The inclusion criteria were randomized controlled trials and observational studies published in English from inception till November 2024. The population of interest was children aged 3 to 17 with unilateral or bilateral alveolar cleft. The material used in surgery was rhBMP-2 and should be compared with the iliac crest for bone reconstruction. Studies were included if they assessed bone formation utilizing cone-beam computed tomography (CBCT) or computed tomography (CT) imaging techniques.

The exclusion criteria included studies that could not be classified as clinical trials or observational studies. Studies on adult patients and patients with previous gingivectomy, without alveolar clefts, or patients with other facial clefts were excluded. Studies that evaluated bone formation using two-dimensional analysis (radiographs) and animals were excluded. Conference proceedings, editorials, review articles, and case series were excluded.

### 2.3. Search Strategy

A comprehensive electronic search was conducted in MEDLINE/PubMed, the Cochrane Central Register of Controlled Trials (CENTRAL), ClinicalTrials.gov (accessed on 10 December 2024), Web of Science, and ScienceDirect for published studies without imposing any time restrictions. The search strategy was designed by one of the authors (E.A.) and approved by the rest of the study team. An amalgamation of medical subject headings (MeSH) terms, such as “Recombinant Human Bone Morphogenetic Protein-2”, “Bone Morphogenetic Protein-2”, “rhBMP-2”, “Alveolar Cleft”, “Cleft Lip and Palate”, and “Maxillary Cleft”, was used to comprehensively identify all studies related to alveolar cleft reconstruction in pediatric patients. Boolean operators (AND/OR) were employed to combine the terms. The manual search for the references for the selected studies was further reviewed to identify any missing articles. The full search strategy is provided in Supplemental Information (Supplementary File S1).

All database articles were identified and exported to Microsoft Excel version 21. (IBM, 365, Washinton, WA, USA). The duplicates were analyzed and removed by Endnote software version 20 (Clarivate, PA, USA). Articles were selected by title and abstract, and finally, the full text was read if the articles followed inclusion criteria. The full texts of all identified studies were then independently screened by five authors (A.S., L.A., M.A., Q.A., and S.A.). Disputes emerging at any stage during the screening process were addressed through discussion and consensus of all authors.

### 2.4. Data Extraction

Five reviewers (A.S., L.A., N.A., R.A., and S.A.) extracted data from the included studies using a pre-designed customized data extraction sheet. The data extracted were author/year/country, study design, number of participants in test and experimental groups, mean age of participants, rhBMP-2 scaffold and dosage, type of alveolar cleft, follow-up, and results. The second data extraction sheet was developed to evaluate the outcomes, such as bone filling, volume, and height, measured in the included studies. The level of evidence for each study was graded by two reviewers (F.O. and Q.A.) using the Oxford Center for Evidence-Based Medicine Scale [13].

### 2.5. Risk of Bias

Four reviewers (A.S., D.A., Q.A., and R.A.) independently assessed the risk of bias in the included studies. The Risk of Bias-2 (RoB-2) tool was used for randomized controlled trials. This tool has three main domains: randomization, sequencing, and reporting. Within each domain are a series of ‘signaling questions’ that aim to evaluate the quality of randomized controlled trials. Judgment in this can be ‘low’, ‘high’, or ‘some concerns’ [14].

For non-randomized trials, the risk of bias in non-randomized studies of interventions (ROBINS-I) tool was used, and three investigators (F.A., E.A., and Q.A.) independently evaluated the risk of bias [15]. This tool has seven domains: selection, confounding, classification of interventions, reporting results, outcomes, missing data, and publication. Each domain is classified as ‘low risk’, ‘high risk’, ‘moderate risk’, and ‘unclear risk of bias’ [15].

### 2.6. Data Synthesis

The primary outcomes assessed were bone filling and volume in the alveolar cleft at three or six months and bone height formation in the alveolar cleft at three, six, nine, and twelve months, both measured using CBCT and CT scanning. The mean bone volume was calculated by subtracting the mean postoperative bone defect volume (mm^3^) from the mean preoperative bone defect volume (mm^3^). Bone height was calculated as the average postoperative bone height formed (mm). Studies comparing rhBMP-2 with iliac crest grafts were pooled for analysis. Results are presented as continuous data using the mean difference (MD), and a forest plot was generated to display the pooled estimates from the meta-analyses. Heterogeneity among the studies was assessed using the I^2^ statistic, with values below 50% indicative of no significant or moderate heterogeneity. The data meta-analysis was performed using RevMan 5.1 software (Cochrane IMS, Copenhagen, Denmark).

### 2.7. GRADE Quality of Evidence

The Grading of Recommendations Assessment, Development, and Evaluation (GRADE) framework was utilized to evaluate the overall quality of evidence from the included studies for each outcome. A “Summary of Findings” table was generated using GRADE software (http://gdt.guidelinedevelopment.org (accessed on 10 December 2024)) [16]. The GRADE methodology assesses the confidence level that the estimated effect or association accurately reflects in the outcome’s actual value. Domains assessed in GRADE depend on the risk of bias, indirectness, imprecision, inconsistency, and publication bias. The result of these domains decides the quality of evidence. The quality of evidence for each domain was categorized as high, moderate, low, or very low [16].

## 3. Results

A total of 430 articles were identified through the electronic database search, from which 55 duplicates were eliminated. Finally, 375 articles were retained to review titles and abstracts, of which 314 were excluded for various reasons, including animal studies, review articles, and studies involving adult populations. A total of forty-three articles were selected for full-text reading, from which thirty-six were excluded for the reason of being review studies and editorials, and articles published in Spanish, French, and Chinese (Supplementary File S2). Finally, seven articles from electronic databases were included in this review.

**Figure 1:**
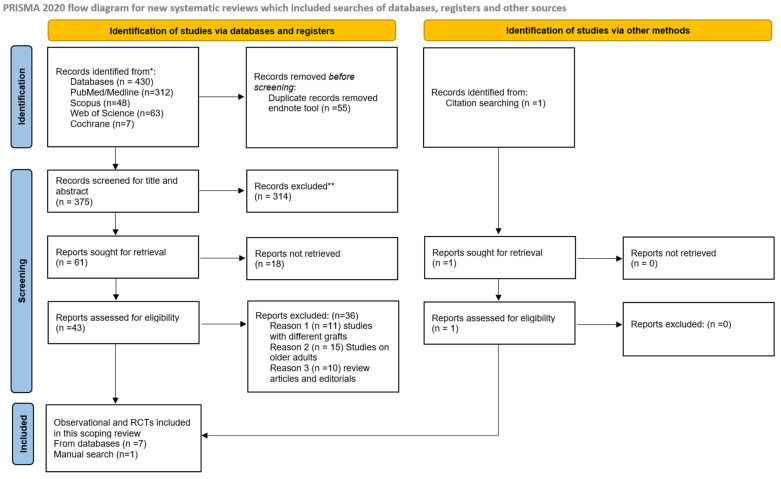
PRISMA flowchart. * articles from different databases searches. ** after reading abstract.

Citation chasing and manual searching of references of the included articles were conducted, and one article was included following this search. Finally, eight articles were included in this review (Figure 1).

### 3.1. Characteristics of Included Studies

The articles included were published between 2006 and 2024 in various countries, such as America, Iran, Brazil, Sweden, and China. Of the eight included studies, six were randomized controlled trials [7,8,17,18,19,20], and two were prospective cohort studies [9,10]; however, due to the characteristics of analysis performed in these studies, they were characterized as non-randomized controlled trials. The average age of patients in the included studies varied between 6 and 14 years, with a mean age of 11.43 + 2.81 years. The number of patients in the control group (iliac crest) and test group (rhBMP-2) varied between 2 and 21, with an average of 9.5 patients.

Two studies by Liang et al. [10] and Harrison et al. [8] included unilateral and bilateral alveolar clefts; however, grafting for bilateral cases was performed in two separate surgical sessions. Across all studies, alveolar cleft reconstruction using rhBMP-2 was compared to iliac crest bone grafts, except in one study where rhBMP-2 was compared with the combination of iliac crest and BMP [8]. The scaffolds used with rhBMP-2 included collagen sponges alone [7,10,17,18,19], collagen sponges combined with demineralized bone tissue [8,9], and hyaluronic acid-based hydrogel [20]. The dosage of rhBMP-2 administered ranged from 250 µg to 4.2 mg.

All the studies had follow-up periods between 3, 6, and 12 months, with an average of 12.7 months. CBCT analyzed the bone volume and height in the maximum studies except for two studies that used CT and convectional occlusal X-rays. Bone quality was not assessed in any of the studies included (Table 1).

**Table 1:** Characteristics of included studies.

Sr No.	Author/Country/Year	Study Design	LOE	Total Number of Patients	Number of Patients in the Test Group (Mean Age)	Number of Patients in the Control Group (Mean Age)	Type of Alveolar Cleft	Type of Iliac Graft	RhBMP-2 Scaffold and Dosage	Follow-Up Duration	Outcome Measuring Instruments	Results
1.	Dickinson et al., 2006, USA [7]	RCT	IIB	n = 21	n = 9; AG = 16.4 ± 1.5 years	n = 12; AG = 15.9 ± 1.9 years	Unilateral cleft	iliac crest cancellous bone (20–30 cc)	S = Absorbable collagen sponge; D = 1.5 mg/mL	6 and 12 months	CBCT	A statistical significance was reported between the groups, with 93% of bone filling in the test group and 63% in the control group.
2.	Alonso et al., 2010, Brazil [17]	RCT	IIB	n = 16	n = 8; AG = 10.5 ± 1.5 years	n = 8; AG = 11.1 ± 0.4 years	Unilateral cleft	iliac crest cancellous bone	S = collagen sponge with lyophilized rhBMP-2; D = 3.2 to 4.2 mg	6 and 12 months	CBCT	No significant difference was reported in bone filling among the groups. While a significant difference was noted in bone height (c = 86.6% and t = 65%).
3.	Canan et al., 2012, Brazil [18]	RCT	IIB	n = 18	n = 6; AG = 9.67 ± 0.5 years	n = 12; AG = 12.12 ± 0.5 years	Unilateral cleft	autologous cancellous bone graft	S = lyophilized rhBMP-2 and absorbable collagen sponge; D = 3.2 to 4.2 mg	3, 6 and 12 months	CBCT	No significant difference was reported between the groups (bone filling-t = 75.1% and c = 78%; bone height = 58%; and 64.4%).
4.	Neovius et al., 2013, Sweden [20]	RCT	IIB	n = 7	n = 2; AG = 9.12 ± 0.5 years	n = 3; AG = 9.7 ± 0.4 years	Unilateral cleft	autologous cancellous bone graft	S = BMP-2-hydrogel; D = 250 mg/mL	6 months	CT	No significant difference was recorded between the groups. Bone density measured after 6 months was t = 46%; c = 48%.
5.	Liang et al., 2017, USA [10]	Prospective Cohort Study	III	n = 35	n = 21; AG = 11.8 ± 1.5 years	n = 12; AG = 11.8 ± 1.5 years	Unilateral and bilateral clefts	iliac crest cancellous bone	S = Absorbable collagen sponge and demineralized bone matrix; D = 2.1 mg	6, 12, and 21 months	CBCT	After 3 months, 67% of patients in the test group and 56% in the control group had complete bone filling. No significant difference was recorded.
6.	Jahanbin et al. 2019, Iran [9]	Prospective cohort study	III	n = 11	n = 6; AG = 12.32 ± 0.9 years	n = 6; AG = 13.12 ± 0.5 years	Unilateral cleft	Cortico-cancellous autogenous bone graft derived from the iliac crest	S = bone morphogenetic proteins (BMPs); D = 0.25 mg	3 and 6 months	CBCT	No statistically significant difference was reported in the groups. However, the study suggests that rhBMP combined with autogenous bone graft could be a better option for treating cleft.
7.	Liu et al., 2020, China [19]	RCT	IIB	n = 26	n = 14; AG = 10.54 ± 1.69 years	n = 12; AG = 11.8 ± 1.5 years	Unilateral cleft	autologous cancellous bone	S = autologous cancellous bone mixed with rhBMP D = 0.5 mg	6 months	CT	A statistically significant bone formation was recorded between the group (*p*-value = 0.022).
8.	Harrison et al., 2024, USA [8]	RCT	IIB	n = 20	n = 10; AG = 10.57 ± 1.72	n = 10; AG = 10.57 ± 1.72	Unilateral and bi-lateral alveolar clefts	Cortico-cancellous autogenous bone graft derived from the iliac crest	S = anterior superior iliac spine with rhBMP-2; D = 1.75 cc:1 cm^3^	6 months	CBCT	In total, 75.9% of bone filling was achieved when the graft was a mixture of iliac spine and rhBMP-2 compared to autogenous bone graft alone.

n = sample size; AG = age group; CBCT = cone beam computed tomography; CT = computed tomography; t = test group; c = control group; S = scaffold; D = dosage; RCT = randomized controlled trials; rhBMP-2 = recombinant human bone morphogenetic protein-2; and LOE = level of evidence.

### 3.2. Bone Filling (Table 2)

The association between bone filling and preoperative bone defect was expressed as mean, standard deviations, and percentage. The average bone volume measured for preoperative analysis in both groups was 1944.28 ± 2087.10 mm^3^ (rhBMP-2) and 1789.10 ± 1980.50 mm^3^ (iliac crest). In the study by Dickinson et al., the average age of patients was higher (16.2 years) compared to other studies; hence, the average measurement of cleft was higher [7]. If this article was removed from the analysis, the average preoperative bone volume would drop significantly to 832 ± 512 mm^3^ in the test group (rhBMP-2) and 917 ± 550.68 mm^3^ in the control group (iliac crest). The percentage of average bone filling in both groups was measured as 61.11% ± 24.6% (test group) and 59.12% ± 18.59% (control group).

**Table 2:** Outcome measures of included studies.

Sr. No	Author	Mean Bone Filling (Test and Control)	Postoperative Bone Filling (Test and Control)	Percentage of Bone Filling (Test and Control)	Mean Bone Height Pre-Op (Test and Control)	Mean Bone Height Post-Op (Test and Control)	Percentage of Bone Height
1.	Dickinson et al. 2006 [7]	N/A	t-not detected; c = 2.0 ± 0.8	t = 95%; c = 63%	N/A	N/A	N/A
2.	Alonso et al., 2010 [17]	t = 974.8; c = 1052.4	t = 247.1; c = 207.8	t = 74.4%; c = 80.4%	t = 15.7; c = 16.1	t = 10.2; c = 13.9	t = 65.0%; c = 86.6%
3.	Canan et al., 2012 [18]	t = 430.4 mm^3^; c = 471.8 mm^3^	t = 354.4; c = 520.5	t = 75.8%; c = 78.0%	N/A	N/A	t = 58.0%; c = 64.2%
4.	Neovius et al., 2013 [20]	N/A	N/A	t = 46%; c = 49%	N/A	N/A	N/A
5.	Liang et al., 2017 [10]	N/A	N/A	t = 78%; c = 66%	N/A	N/A	t = 31.6%; c = 32.0%
6.	Jahanbin et al., 2019 [9]	t = 13.3 ± 0.6; c = 14.2 ± 3.6	t = 366.8 ± 42.9; c = 438.2 ± 135.6	N/A	t = 12.9 ± 0.89; c = 14.1 ± 3.2	t = 12.9 ± 1.1; c = 10.9 ± 3.6	N/A
7.	Liu et al., 2020 [19]	N/A	t = 0.52 ± 0.20 cm^3^; c = 0.69 ± 0.21 cm^3^	t = 42.01 ± 15.57%; c = 55.79 ± 11.84%	N/A	N/A	N/A
8.	Harrison et al., 2024 [8]	N/A	t = 7.66 ± 1.20 mm^3^; c = N/A	t = 75.9%; c = N/A	t = 469.22 ± 217.44 mm^3^	t = 109.40 ± 77.0 mm^3^	t = 75.87 ± 9.52%

t = test group; c = control group; and N/A = not applicable.

### 3.3. Bone Height (Table 2)

The association between pre and postoperative bone height of alveolar clefts was measured in percentages. The bone height was measured in three articles [10,17,18], with a preoperative mean of 16.5 ± 1.67 mm in the rhBMP-2 group and 17.8 ± 1.48 mm in the iliac group. The overall percentage of bone height measured in three studies was 67.5% ± 5.45% in the rhBMP-2 group and 78.65% ± 14.38% in the iliac crest group.

### 3.4. Risk of Bias (Figure 2 and Figure 3)

Four RCTs included in this review have a low risk of bias; however, two included RCTs have some concerns. All the included studies have at least one domain classified as high-risk. In two articles, the blinding of participants was at a low risk, as the surgeons picked participants according to their 3D analysis [7,18]. Three articles were not sure about the randomization of the participants [7,17,20]. Four articles did not measure bone height in their results [7,8,19,20], even though it was mentioned as an outcome of the study.

Regarding selective reporting of results, no study had a pre-registered protocol, which precluded the determination of whether any additional outcomes were omitted from publication. However, the variables outlined in the Section 2 were consistently reported in the Section 3, justifying their classification as having a low risk of bias. However, all the included studies presented their results and statistical analysis according to the CONSORT guidelines.

Both the included non-randomized trials had a moderate risk of bias. The domain of random sequencing and allocation concealment was classified at higher risk in the study by Liang et al., as the authors allowed the parents of patients to choose between both interventions [10].

**Figure 2:**
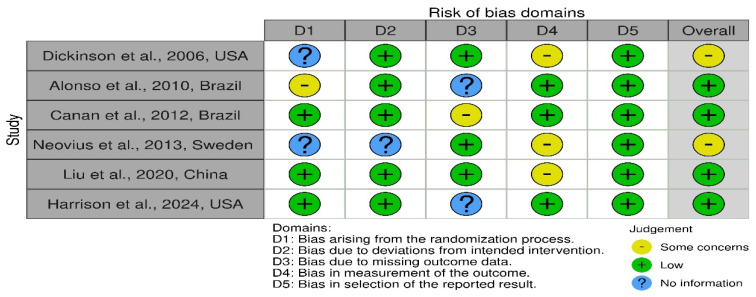
Risk of bias for randomized control trials (RoB-2 tool) [7,8,17,18,19,20].

**Figure 3:**
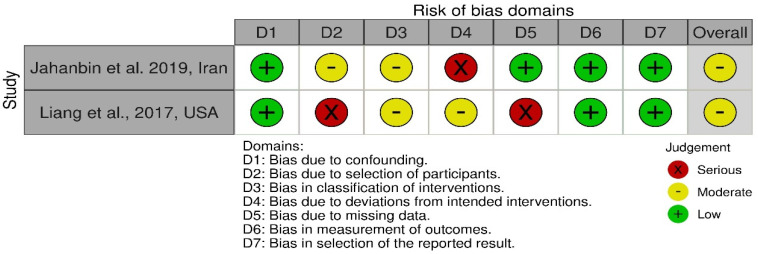
Risk of bias for non-randomized control trials (ROBINS-I) [9,10].

### 3.5. Meta-Analysis (Figure 4)

In the bone filling and formation analysis, five studies were compared [8,17,18,19,20]. There is no statistically significant difference between the test and control groups, according to the analysis conducted using the random effects model and the inverse variance method to compare the standardized mean difference (SMD); the summarized standardized mean difference (SMD) measured was −1.24, with a 95% confidence interval ranging between −4.14 and 1.67. More significant bone formation was reported in the rhBMP-2 group than in the control group. The test for the overall effect does not show a significant effect. A significant heterogeneity was present (*p* < 0.01), signifying varying effects in extent and/or direction. An I^2^ indicates that 89% of the cohort differences arise from heterogeneity rather than arbitrary chance. The lack of data made it challenging to perform subgroup and meta-analysis for the outcome related to bone height.

**Figure 4:**
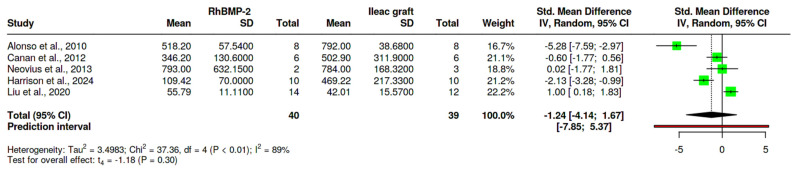
Forest plot of a pooled analysis of the mean bone filling and bone height [8,17,18,19,20]. Green color represents confidence interval.

### 3.6. GRADE Quality of Evidence (Table 3)

The quality of evidence of the five articles included in the meta-analysis was assessed with GRADE analysis. All these articles were RCTs; hence, the quality of evidence was reported as high. However, the methodological quality of the included studies was moderate due to the randomization of participants and the serious risk of bias. In the GRADE analysis, the risk of bias was considered downgraded due to randomization of participants and the surgeons. Secondly, due to allocation concealment, analysis of results, and statistical analysis. The inconsistency of the included RCTs was not serious since methodological quality and clinical performance were acceptable. Indirectness was also not serious, as the evidence regarding population, intervention, comparison, and outcome was clearly defined and answered. Impression was downgraded in this review due to the smaller number of participants. Publication bias was also not serious, as no pre-defined protocol was mentioned in any included studies. Overall, the evidence reported was moderate, emphasizing that rhBMP-2 may improve bone regeneration when used for cleft surgeries. However, more RCTs with larger sample sizes and follow-ups are required to support the findings of the current review.

**Table 3:** GRADE quality of evidence for included RCTs.

RhBMP-2 Graft Compared to Ileac Crest for Bone Regeneration in Alveolar Cleft of Children											
**Bibliography:** *population: children with unilateral or bilateral cleft; intervention: rhBMP-2 graft alone or in conjunction with other grafts; and comparison: ileac crest; outcome: bone filling, height, and volume difference among both the groups.*											
**Certainty Assessment**							**Summary of Findings**				
**Participants (Studies) Follow-Up**	**Risk of Bias**	**Inconsistency**	**Indirectness**	**Imprecision**	**Publication Bias**	**Overall Certainty of Evidence**	**Study Event Rates (%)**		**Relative Effect (95% CI)**	**Anticipated Absolute Effects**	
							**With Ileac Crest**	**With RhBMP-2 Graft**		**Risk with Ileac Crest**	**Risk Difference with RhBMP-2 Graft**
Bone filling (mm^3^) (follow-up: range 3 months to 12 months; assessed with: CBCT)											
79 (5 RCTs)	serious ^a^	not serious	not serious	serious ^b^	all plausible residual confounding would suggest spurious effect, while no effect was observed	⨁⨁⨁◯ Moderate ^a,b^	40/79 (50.6%)	39/79 (49.4%)	RR −4.14 (−4.14 to 1.67)	The mean bone filling was 697.92 mm^3^	MD was 210.78 mm^3^ lower than 165.93 (from 100 fewer to 30 more) ^c^

CI: confidence interval; RR: risk ratio; MD: mean difference. GRADE evidence explanation: very low: very little confidence in effect and estimate of the outcome; low: low certainty implies that the available evidence is limited, and the true effect may be substantially different from the estimate; moderate: moderate certainty suggests that the available evidence is sufficient to support a conclusion, but further research may still impact confidence in the estimate; high: high certainty indicates that the available evidence provides a high level of confidence in the estimate of the effect. Explanations: ^a^. The included studies lack blinding of participants and the surgeons performing the surgery. The blinding of participants was performed only in one study. ^b^. The included studies have a low number of participants. Hence, the impression is low. ^c^. The risk of intervention group is based on the assumed risk of comparison group.

## 4. Discussion

The meta-analysis and GRADE quality assessment results support the hypothesis proposed in this systematic review. Although literature generally favors using rhBMP-2 over the iliac crest for alveolar reconstruction, the studies included in this review yielded mixed results. The meta-analysis found no significant difference between the treatments after 6 to 12 months of follow-up. Notably, most articles included were randomized controlled trials with high-quality evidence. However, heterogeneity was observed in terms of protein dosage, scaffold materials, follow-up periods, and assessed outcomes. Although evidence underscores the benefits of bioactive materials in bone regeneration and the reconstruction of cleft lip and palate defects, further investigation is required to determine the most effective protein type and dosage. Additionally, research should focus on elucidating the interactions between bioactive materials and other proteins and examining their temporal dynamics and releasing kinetics.

The classical tissue engineering triangle must be present for tissue regeneration: a signal for bone formation, a source of cells, and a matrix [21]. The U.S. Food and Drug Administration approved using rhBMP-2 in human spine fusion procedures [2,22]. The clinical application of bone morphogenetic proteins began with their use in spinal fusion and the treatment of nonunion fractures within orthopedic practices [23]. Studies reported that rhBMP-2 is the best alternative for vertebral and maxillofacial fractures [23,24]. Moreover, this bioactive material attracts the osteoprogenitor and stem cells of the body to form new bone cells.

Most clinical trials in this study used absorbable collagen sponges (ACS) as the scaffold material for delivering rhBMP-2, a system approved by the U.S. Food and Drug Administration [25]. This delivery method facilitates the attachment of osteoblasts to the defective bone and allows for scaffold resorption within 4 to 12 weeks postoperatively [25]. Absorbable collagen sponge (ACS) is biocompatible and degrades into physiologically compatible compounds, making it well-suited for interactions with cells and macromolecules. Notably, ACS retains 95% of rhBMP-2 after 15 min of soaking [26,27]. However, a key limitation identified in the reviewed studies is its inability to regulate the release kinetics of BMP-2, often necessitating higher rhBMP-2 dosages to achieve the desired therapeutic effects [11,28]. Enhancing carrier systems with improved structural stability and controlled release mechanisms could optimize bone induction by ensuring sustained protein availability to local osteoprogenitor cells during new bone formation.

Norvis et al. investigated using a hyaluronan-based hydrogel to enhance the efficacy of BMP-2. This hydrogel demonstrated improved control over BMP-2 release compared to absorbable collagen sponges (ACS), which is attributed to the electrostatic interactions between hyaluronan and bioactive molecules [20]. The study found that low BMP-2 concentrations (50 mg/mL) did not promote bone formation after six months. However, increasing the BMP-2 concentration to 250 mg/mL significantly enhanced bone formation, yielding favorable outcomes [20].

The age group included in the current review is between 6 and 15 years, in which bone graft healing is highly successful. Studies reported that after the eruption of permanent dentition, the outcome of the surgery is highly unpredictable. The mean age of patients in this review was 11.48 ± 1.91; however, if the article included young adults eliminated, the average age range would be 10.58 ± 1.00 years. These findings suggest that the current study’s participants represent the best age group to perform alveolar cleft reconstruction surgery.

The current review reported high diversity in preoperative bone defect with an average of 1944.28 ± 2087.10 mm^3^ (rhBMP-2) and 1789.10 ± 1980.50 mm^3^ (iliac crest). However, after excluding the study by Dickinson et al., which included adult patients, the average palatal bone defect reduced to 832 ± 512 mm^3^ in the test group (rhBMP-2) and 917 ± 550.68 mm^3^ in the control group (iliac crest) [7]. This variation results from changes in the alveolar cleft volume of adult patients, and they have less bone regeneration. None of the included studies evaluated the defect size of the patients recruited in the rhBMP-2 and iliac crest groups. It would be interesting to record the defect size before the surgery and evaluate the efficacy of bone graft in reconstruction. Conversely, the result from Dickinson et al. supported the use of iliac crest graft (MD: 2100.00 [CI: 1865.29 to 2334.71]) in bone reconstruction surgery. This study concluded that in the broader clefts, the iliac bone graft provides a desirable outcome compared to rhBMP-2 [7]. However, this analysis should be interpreted cautiously, as all the other studies either favored rhBMP-2 or had similar results with both groups.

Six studies examined unilateral cleft [7,9,17,18,19,20], and two studied both unilateral and bilateral clefts [8,10]. The nature of reconstruction varied among the types of clefts since they have differences in irrigation and stability. As a result, even though bilateral surgery requires two sets of surgeries, the local conditions varied between the two surgeries. Studies evaluated the graft remaining in both unilateral and bilateral surgeries and reported that 70% of grafts remain in unilateral patients and 45% in bilateral patients [29,30,31].

Studies reported that the dosage directly influences the osteoinductive property of rhBMP-2 [21,32,33]. For instance, low concentrations of bioactive protein primarily result in the formation of significant cartilage tissue with minimal bone tissue. Therefore, the concentration of rhBMP-2 at the graft site is more important than the total dose administered. The studies included in this review showed considerable variation in rhBMP-2 dose volume, ranging from 250 mg/mL to 4.2 mg. Neovius et al. employed a small hydrogel scaffold, as one objective of their study was to reduce the dosage and assess its efficacy [20]. If this study were excluded from the systematic review, the rhBMP-2 dosage across the remaining studies would average between 1.5 and 4.2 mg. However, none of the included studies evaluated the dose–response relationship concerning cleft volume and scaffold size.

Several side effects, such as ectopic bone formation, vertebral osteolysis, and post-operative radiculitis have been reported with the use of rhBMP-2 [34]. In 2008, the FDA made a declaration that there is a possibility of retropharyngeal edema with the use of rhBMP-2 in cervical spinal fusion [34]. However, no serious complication was reported with the use of rhBMP-2 in maxillofacial surgeries.

The follow-up period in the included studies ranged from 6 to 21 months, which might cause some concerns. It is recommended that bone formation be evaluated for between 4 and 6 months, during which integrated bone implant installation is completed. If the newly formed bone tissue is not under-functioning at the 21-month follow-up, it remodels and recovers [23].

Cone-beam computed tomography is the best imaging technique since it is more precise and repeatable than traditional X-rays [24]. In all studies included in the analysis, the evaluation of bone volume was conducted using a CBCT imaging technique. All studies indicated that the bone formation volume in the experimental group was equal to or greater than that observed in the control group.

We encourage and anticipate that future studies should be carried out surrounding the findings of current systems. In this study, the complete focus was on the use of rhBMP-2 in alveolar surgery; however, future studies should focus on other bioactive materials such as bovine hydroxyapatite, Bio-Oss, and autologous bone graft. Based on the data studied, the use of rhBMP-2 is advised in alveolar bone reconstruction. However, the counseling of potential adverse outcomes and long-term consequences should be discussed with the patients. One limitation of the current review is its focus on rhBMP-2 and ileac bone graft; hence, future studies should compare other bone grafts with rhBMP-2 to advise more efficient and effective alternatives for alveolar bone reconstruction. Another limitation of the study is the focus on bone height and volume; future studies should try to incorporate parameters such as healing time, long-term stability, and patient-related outcomes.

## 5. Conclusions

Within the limitations of this systematic review and meta-analysis, it can be concluded that rhBMP-2 has the potential to serve as one of the best alternatives for alveolar bone reconstruction surgeries. The GRADE analysis and meta-analysis results suggest a moderate likelihood that rhBMP-2 could be an effective alternative for such procedures. However, a definitive conclusion cannot be drawn due to the limited number of studies. Therefore, further randomized trials and clinical studies with larger sample sizes and longer follow-up durations are necessary to substantiate the findings of this review. Additionally, performing subgroup analyses based on cleft volume in future research would be valuable.

## Data Availability

On request from corresponding author.

## Supplementary Materials

The following are available online at https://www.mdpi.com/article/10.3390/life15020185/s1, File S1: PRISMA checklist, articles excluded with reasons, past systematic review on similar topics with limitations. File S2: Reason for exclusion. File S3: Previous systematic review summary.
