# Supplementary material for: Efficacy of Recombinant Human Bone Morphogenetic Protein-2 in Alveolar Cleft Treatment for Children: Systematic Review and Meta-Analysis
Source: Life (Basel). 2025 Jan 26;15(2):185. doi: 10.3390/life15020185 (PMC11856092; doi:10.3390/life15020185)
Supplement: Supplementary file 1 [file life-15-00185-s001.zip › Supplemantry file S3.pdf]

Supplementary File S3 - Previous systematic review summary

Author	Search end date	Research question/s	Databases searched	Participant	Inclusion and exclusion criteria	Limitations
Uribe et al., [1]	Not reported	Not reported	Medline, EMBASE, Central Cochrane, LILACS, Cinahl, and SCOPUS	Participants of any age group with cleft lip and palate	Inclusion<br>- Human studies<br>- English and Spanish<br>- Abstract Available<br>- Adolescent and adult as subject.<br>- No time limitation<br>- RCTs<br>- Comparison of rhBMP-2 with iliac bone graft<br>Exclusion<br>- Non-human<br>- Review<br>- No abstract<br>- Case studies, report, opinion and qualitative	- Does not performed citation chasing<br>- Date of search unknown<br>- Didn't specify limitations of outcomes<br>- Unclear search strategy and search terms (no use of Boolean operators and wild cards)<br>- Vague interpretation of results "high" – no numerical analysis from the studies.<br>- Poor reporting, unable to replicate study.<br>- Failed to mention the criteria for level of evidence
Liang, et al. [2]	Not reported	Not reported	Medline/PubMed and Google Scholar	Participants from all age group	Inclusion<br>- alveolar augmentation, alveolar socket grafting, and alveolar cleft repair within the oral and maxillofacial.<br>- No language restriction<br>Exclusion<br>Not mentioned	- Limited sample size<br>- No meta-analysis was performed.<br>- Methodological errors<br>- Did not followed PRISMA guidelines

Rosa et al., [3]	Inception till 2018	Not reported	PubMed (Medline), Lilacs, Ibecs, Web of Science, BBO, Scopus, and The Cochrane Library.	Participants from all age group.	Inclusion<br>- peer-reviewed scientific literature published in English.<br>- retrospective or prospective clinical trials<br>- studies that evaluated the use of bioactive proteins in the treatment of patients with cleft lip and palate defects.<br><br>Exclusion<br>- Review articles, in vitro studies, case series or case reports, and clinical trials without a control group.	- No GRADE analysis was performed<br>- Inclusion criteria was not clearly defined<br>- Risk of bias tool not mentioned and reported clearly<br>- Publication bias.<br>- Subgroup analysis has not been clearly explained<br>- No conclusion and limitations were given.

References

1. Uribe, F.; Alister, J.P.; Zaror, C.; Olate, S.; Farina, R. Alveolar cleft reconstruction using morphogenetic protein (rhBMP-2): A systematic review and meta-analysis. *The Cleft Palate-Craniofacial Journal* **2020**, *57*, 589-598.
2. Liang, F.; Leland, H.; Jedrzejewski, B.; Auslander, A.; Maniskas, S.; Swanson, J.; Urata, M.; Hammoudeh, J.; Magee III, W. Alternatives to autologous bone graft in alveolar cleft reconstruction: the state of alveolar tissue engineering. *Journal of Craniofacial Surgery* **2018**, *29*, 584-593.
3. da Rosa, W.L.d.O.; da Silva, T.M.; Galarça, A.D.; Piva, E.; da Silva, A.F. Efficacy of rhBMP-2 in cleft lip and palate defects: systematic review and meta-analysis. *Calcified Tissue International* **2019**, *104*, 115-129.
