# Supplementary material for: Efficacy of Recombinant Human Bone Morphogenetic Protein-2 in Alveolar Cleft Treatment for Children: Systematic Review and Meta-Analysis
Source: Life (Basel). 2025 Jan 26;15(2):185. doi: 10.3390/life15020185 (PMC11856092; doi:10.3390/life15020185)
Supplement: Supplementary file 1 [file life-15-00185-s001.zip › Supplementary file S2.pdf]

Supplementary File S2: Reason for exclusion

Sr. No.	Article	Reason for exclusion
1.	Alternatives to Autologous Bone Graft in Alveolar Cleft Reconstruction: The State of Alveolar Tissue Engineering	Review article
2.	Management of failed alveolar bone grafts: improved outcomes and decreased morbidity with allograft alone	Not on efficacy of rhBMP-2
3.	Pivotal, randomized, parallel evaluation of recombinant human bone morphogenetic protein-2/ absorbable collagen sponge and autogenous bone graft for maxillary sinus floor augmentation.	Maxillary sinus surgery study
4.	De novo bone induction by recombinant human bone morphogenetic protein-2 (rhBMP-2) in maxillary sinus floor augmentation.	Maxillary sinus surgery
5.	A prospective, randomized, controlled, multicenter evaluation of extraction socket preservation comparing two bovine xenografts: clinical and histologic outcomes	Extraction surgery
6.	Comparison of bovine-derived hydroxyapatite and autogenous bone for secondary alveolar bone grafting in patients with alveolar clefts.	Comparison of different materials
7.	A comparative analysis of rhBMP-2/DBM vs. ICBG for secondary alveolar bone grafts in patients with cleft lip and palate: review of 501 cases.	Case series
8.	Recombinant human bone morphogenetic protein type 2 application for a possible treatment of bisphosphonates-related osteonecrosis of the jaw.	Other jaw surgeries
9.	Immediate distraction osteogenesis: the sandwich technique in combination with rhBMP-2 for anterior maxillary and mandibular defects.	Other facial surgeries
10.	Systematic review and meta analysis of recombinant human bone morphogenetic protein-2 in localized alveolar ridge and maxillary sinus augmentation	Review article
11.	Use of recombinant human Bone Morphogenetic Protein (rhBMP-2) in reconstruction of maxillary alveolar clefts.	Adult patients
12.	Higher dosages of BMP-2 in alveolar cleft repair result in higher rates of postoperative nasal stenosis.	Nasal reconstruction
13.	Randomized study evaluating recombinant human bone morphogenetic protein-2 for extraction socket augmentation.	Socket augmentation
14.	Horizontal ridge augmentation of the atrophic anterior maxilla using rhBMP-2/ACS or autogenous bone grafts: a proof-of-concept randomized clinical trial.	Ridge augmentation
15.	Bone morphogenetic protein induced repair of the premaxillary cleft.	Case report
16.	Primary reconstruction of alveolar clefts using recombinant human bone morphogenic protein-2: clinical and radiographic outcomes.	Radiographic analysis
17.	The clinical application of rhBMP-7 for the reconstruction of alveolar cleft.	Not comparison study
18.	Nationwide practice patterns in the use of recombinant human bone morphogenetic protein-2 in pediatric spine surgery as a function of patient-, hospital-, and procedure-related factors.	Survey study

19.	Effectiveness and harms of recombinant human bone morphogenetic protein-2 in spine fusion: a systematic review and meta-analysis.	Review article
20.	Safety and effectiveness of recombinant human bone morphogenetic protein-2 for spinal fusion: a meta-analysis of individual-participant data.	Review article
21.	Alveolar Cleft Reconstruction Using Morphogenetic Protein (rhBMP-2): A Systematic Review and Meta-Analysis	Review article
22.	The presurgical status of the alveolar cleft and success of secondary bone grafting	Not comparison
23.	Management of alveolar clefts.	Other techniques
24.	Bone morphogenetic proteins: structure, biological function and therapeutic applications.	Application of rhBMP-2
25.	Alveolar bone grafting: a review of 115 patients	Review article
26.	Bone morphogenetic proteins in craniomaxillofacial surgery.	Craniofacial surgeries
27.	Secondary bone grafting for alveolar cleft in children with cleft lip or cleft lip and palate.	Review article
28.	Regenerative medicine in the treatment of alveolar cleft defect: a systematic review of the literature.	Review article
29.	Delivering on the promise of bone morphogenetic proteins.	Application of rhBMP-2
30.	Alveolar reconstruction in cleft patients: decreased morbidity and improved outcomes with supplemental demineralized bone matrix and cancellous allograft.	No comparison
31.	Cost-effectiveness analysis of demineralized bone matrix and rhBMP-2 versus autologous iliac crest bone grafting in alveolar cleft patients	Cost-effectiveness
32.	Cleft palate reconstruction using collagen and nanofiber scaffold incorporating bone morphogenetic protein in rats.	Animal study
33.	Alveolar bone healing accompanied by severe swelling in cleft children treated with bone morphogenetic protein-2 delivered by hydrogel.	Conference proceedings
34.	Delivering on the promise of bone morphogenetic proteins	Application of rhBMP-2
35.	Bone morphogenetic proteins: basic concepts	Application of rhBMP-2
36.	Efficacy of rhBMP-2 in Cleft Lip and Palate Defects: Systematic Review and Meta-analysis	Review article
